# Supplementary material for: Synthesis and Characterization of Nano-Sized 4-Aminosalicylic Acid–Sulfamethazine Cocrystals
Source: Pharmaceutics. 2021 Feb 19;13(2):277. doi: 10.3390/pharmaceutics13020277 (PMC7923100; doi:10.3390/pharmaceutics13020277)
Supplement: Supplementary file 1 [file pharmaceutics-13-00277-s001.pdf]

pharmaceutics

Supplementary Materials: Synthesis and Characterization of Nano-Sized 4-Aminosalicylic Acid–Sulfamethazine Cocrystals

Ala' Salem, Anna Takácsi-Nagy, Sándor Nagy, Alexandra Hagymási, Fruzsina Gősi, Barbara Vörös-Horváth, Tomislav Balić, Szilárd Pál and Aleksandar Széchenyi

Dissolution Test

UV-VIS Absorption Spectra in Aqueous Solution

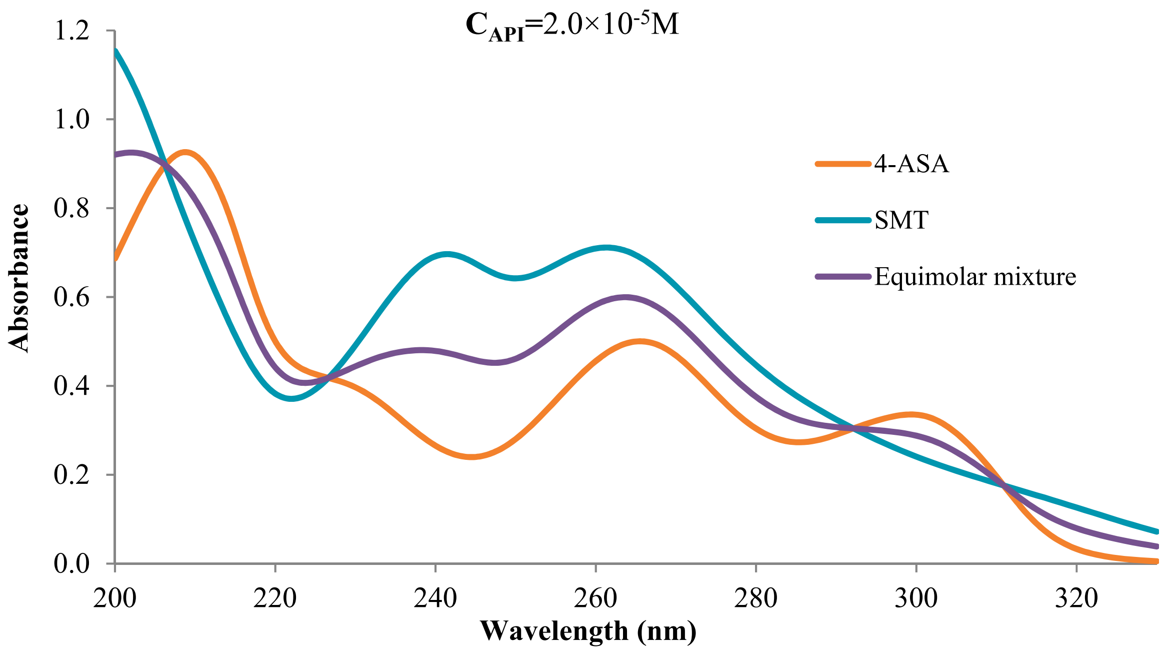

Figure S1. Spectral properties of 4-ASA, SMT, and their equimolar mixture, no interaction of drugs accrued in a water solution.

Calibration

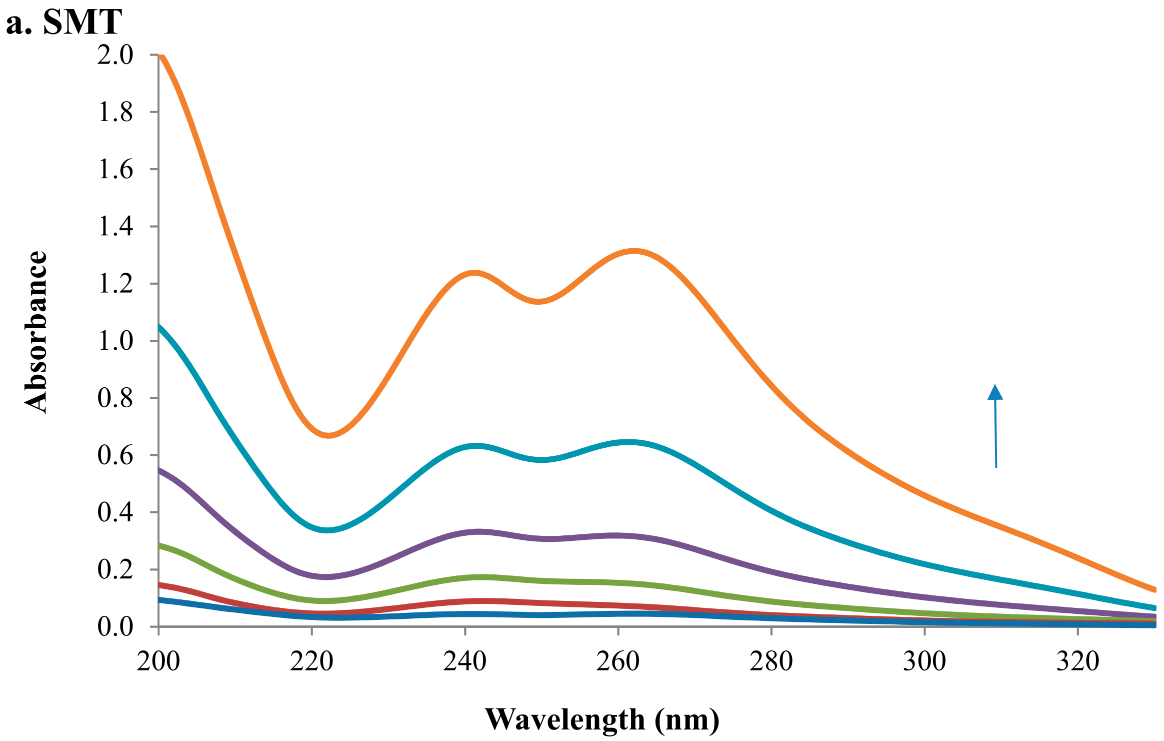

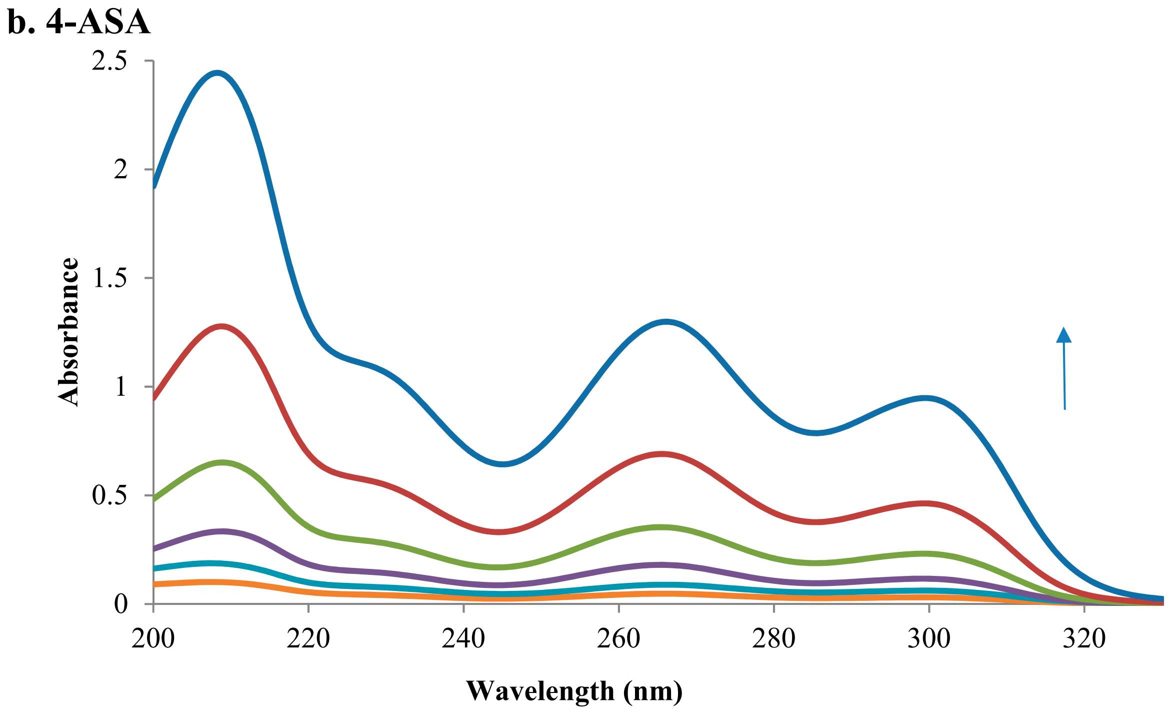

Figure S2. Absorbance of SMT (a) and 4-ASA (b) at different concentrations.

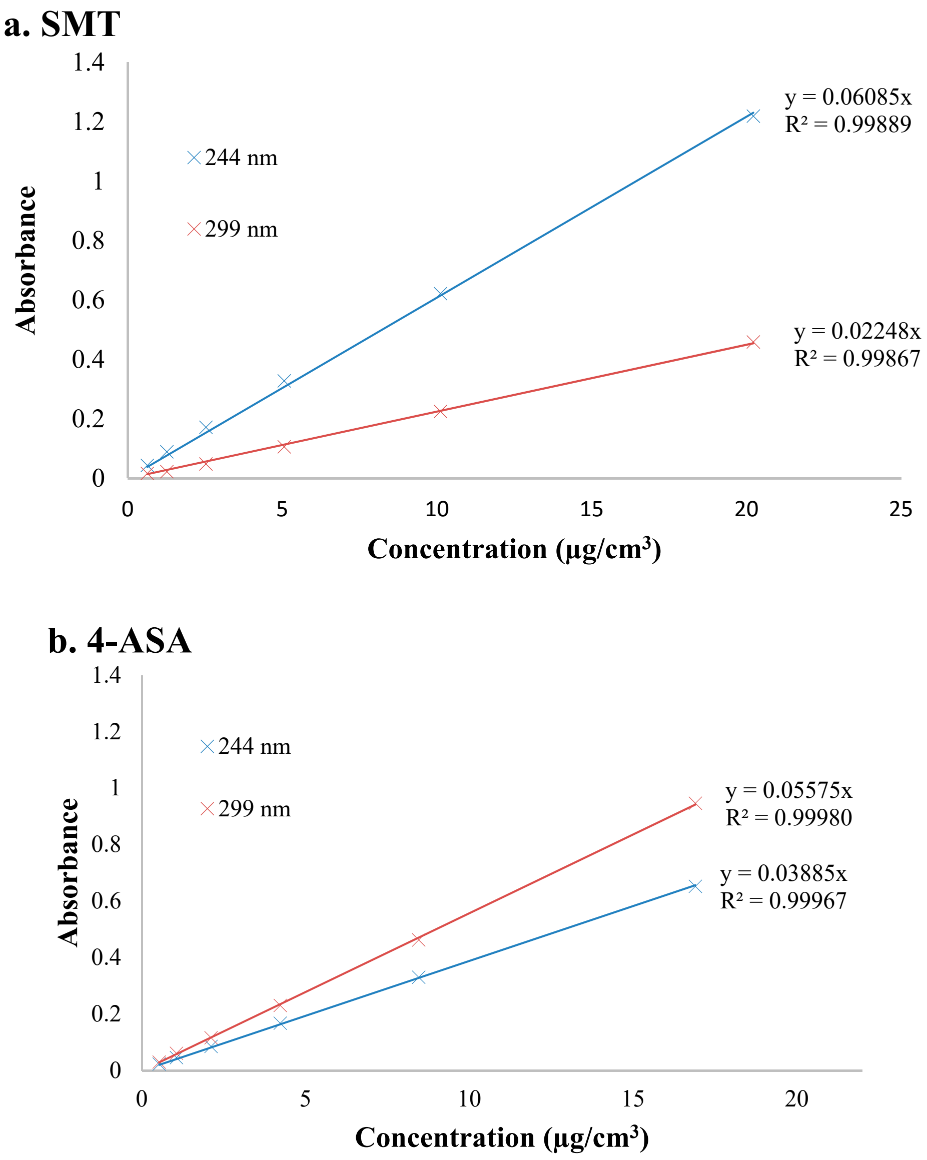

Figure S3. Calibration curves of SMT (a) and 4-ASA (b) at 244 and 299nm.

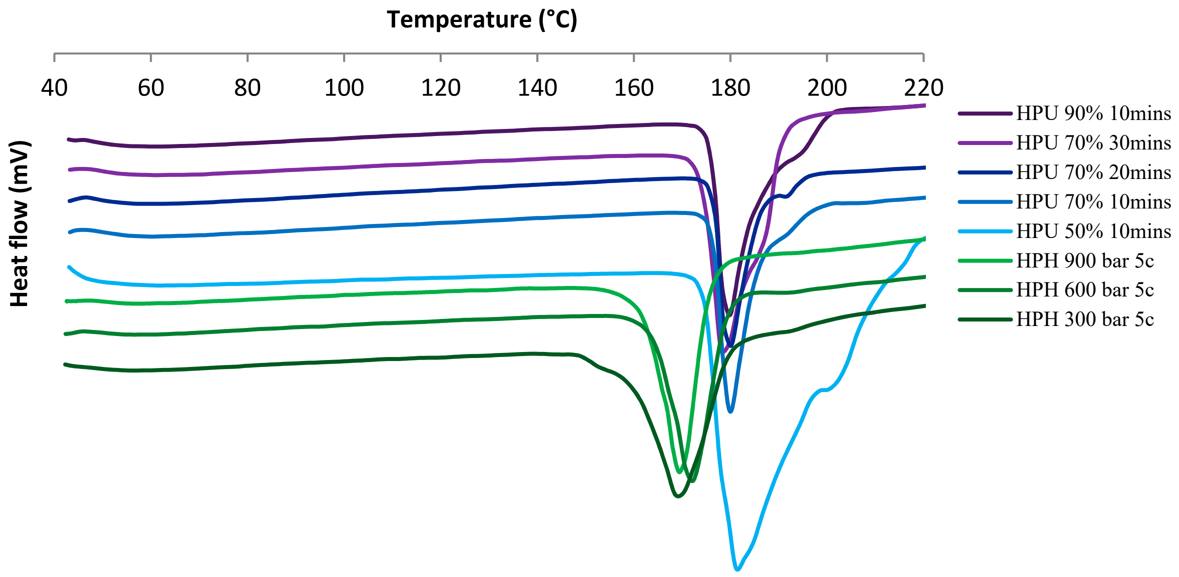

Figure S4. DSC thermograms of the co-crystals. HPH: high pressure-homogenization; HPU: high-power ultrasound; c: number of HPH cycles; percentage refers to HPU amplitude.

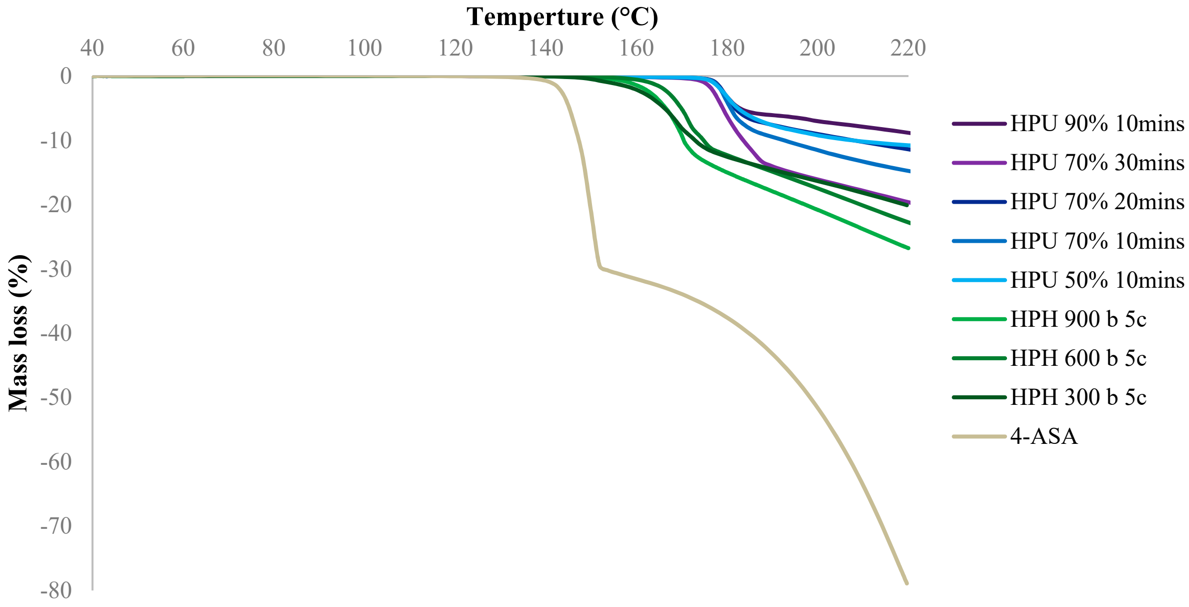

Figure S5. TGA plot of the co-crystals and 4-ASA HPH: high pressure-homogenization; HPU: high-power ultrasound; c: number of HPH cycles; percentage refers to HPU amplitude.

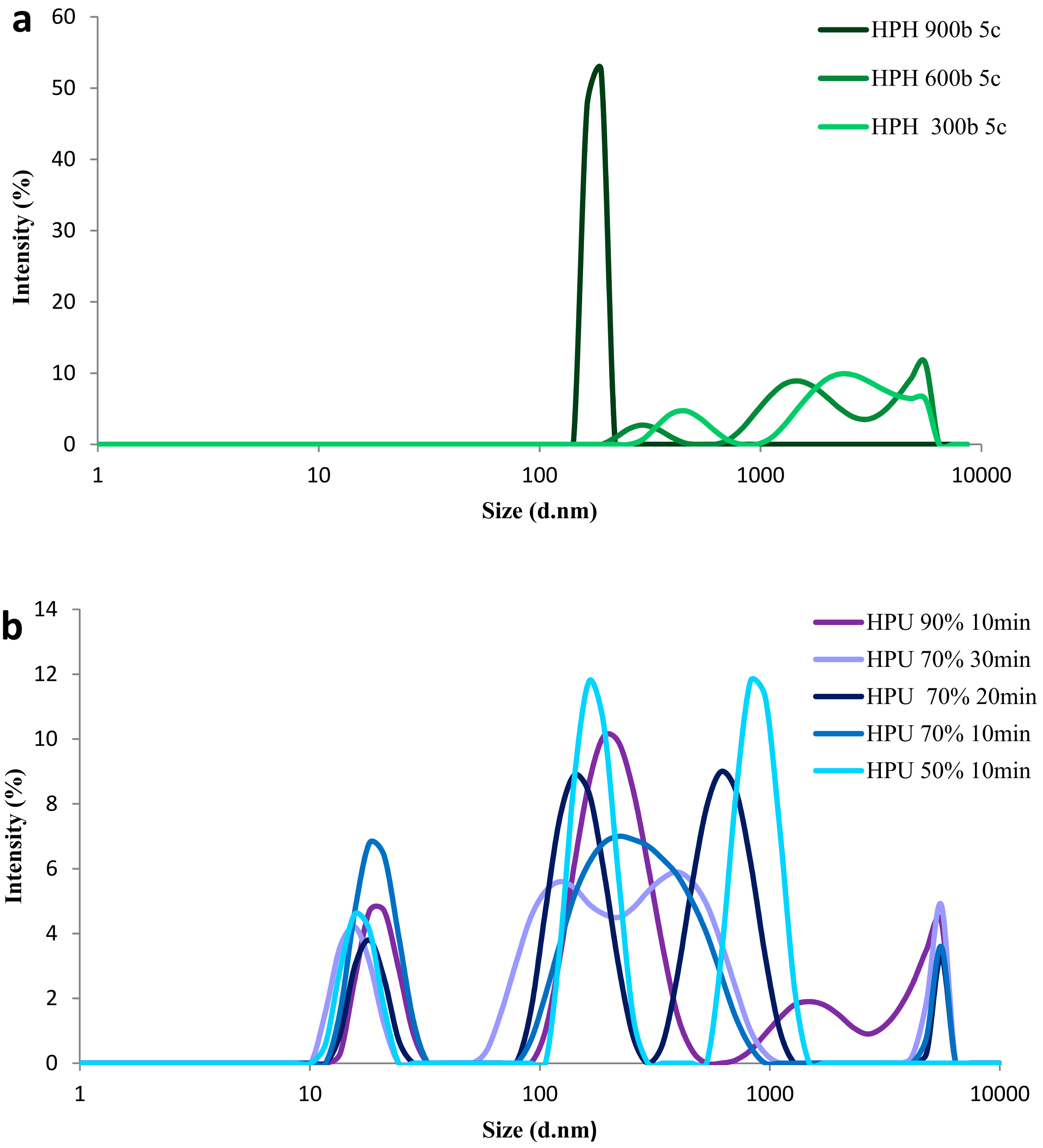

Figure S6. DLS size distribution by intensity (**a**) cocrystals prepared by HPH; (**b**) cocrystals prepared by HPU. HPH: high pressure-homogenization; HPU: high-power ultrasound; c: number of HPH cycles; percentage refers to HPU amplitude.

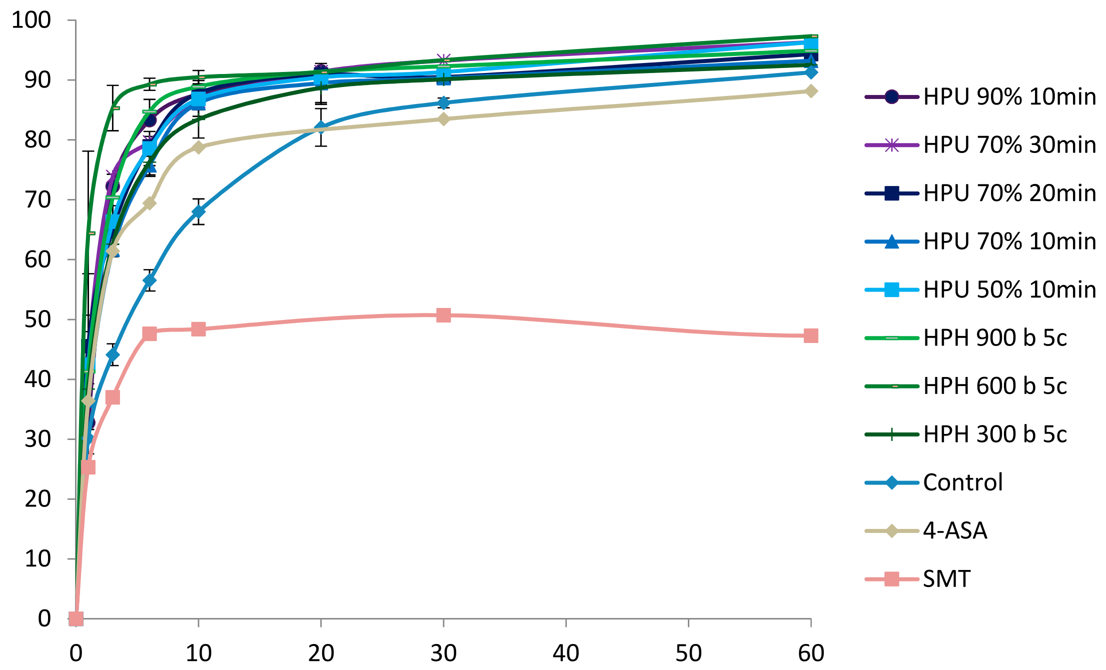

Figure S7. SMT dissolution from the co-crystals and dissolution of pure APIs and control. HPH: high pressure-homogenization; HPU: high-power ultrasound; c: number of HPH cycles; percentage refers to HPU amplitude.

Table S1. SMT absorbance measurements at 244 and 299 nm.

**SMT**	**244 nm**	***n*=3**	**299 nm**	***n*=3**
µg/mL	Mean	SD	Mean	SD
0.63	0.0436	0.0016	0.0166	0.0048
1.26	0.0894	0.0035	0.0226	0.0008
2.53	0.1719	0.0062	0.0484	0.0039
5.06	0.3283	0.0097	0.1059	0.0098
10.11	0.6215	0.0218	0.2250	0.0298
20.22	1.2188	0.0190	0.4590	0.0183

Table S2. 4-ASA absorbance measurements at 244 and 299 nm.

**4-ASA**	**244**	***n*=3**	**299 nm**	***n*=3**
µg/mL	Mean	SD	Mean	SD
0.53	0.0239	0.0030	0.0308	0.0019
1.06	0.0463	0.0023	0.0617	0.0055
2.11	0.0863	0.0006	0.1166	0.0007
4.23	0.1685	0.0004	0.2314	0.0003
8.45	0.3311	0,0012	0.4629	0.0014
16.90	0.6464	0.0131	0.9472	0.0147

Table 3. Hydrodynamic diameter of the samples.

**Sample**	**Z-Average (d.nm)**
Control	274.7
HPH 300 bar 5c	1813
HPH 600 bar 5c	1953
HPH 900 bar 5c	8014
HPU 50% 10min	473.9
HPU 70% 10min	531.2
HPU 70% 20min	331.6
HPU 70% 30min	214.2
HPU 90% 10min	205.9
